# Nanoarchitectonics of Sustainable Food Packaging: Materials, Methods, and Environmental Factors

**DOI:** 10.3390/ma18051167

**Published:** 2025-03-06

**Authors:** Tangyu Yang, Andre G. Skirtach

**Affiliations:** Nano-Biotechnology Group, Faculty of Bioscience Engineering, Ghent University, 9000 Ghent, Belgium; tangyu.yang@ugent.be

**Keywords:** nanoarchitectonics, nanotechnology, food, packaging, sustainable, materials, polymers, microplastics

## Abstract

Nanoarchitectonics influences the properties of objects at micro- and even macro-scales, aiming to develop better structures for protection of product. Although its applications were analyzed in different areas, nanoarchitectonics of food packaging—the focus of this review—has not been discussed, to the best of our knowledge. The (A) structural and (B) functional hierarchy of food packaging is discussed here for the enhancement of protection, extending shelf-life, and preserving the nutritional quality of diverse products including meat, fish, dairy, fruits, vegetables, gelled items, and beverages. Interestingly, the structure and design of packaging for these diverse products often possess similar principles and methods including active packaging, gas permeation control, sensor incorporation, UV/pulsed light processing, and thermal/plasma treatment. Here, nanoarchitechtonics serves as the unifying component, enabling protection against oxidation, light, microbial contamination, temperature, and mechanical actions. Finally, materials are an essential consideration in food packaging, particularly beyond commonly used polyethylene (PE), polypropylene (PP), polyethylene terephthalate (PET), polystyrene (PS), and polyvinyl chloride (PVC) plastics, with emphasis on biodegradable (polybutylene succinate (PBS), polyvinyl alcohol (PVA), polycaprolactone (PCL), and polybutylene adipate co-terephthalate (PBAT)) as well as green even edible (bio)-materials: polysaccharides (starch, cellulose, pectin, gum, zein, alginate, agar, galactan, ulvan, galactomannan, laccase, chitin, chitosan, hyaluronic acid, etc.). Nanoarchitechnotics design of these materials eventually determines the level of food protection as well as the sustainability of the processes. Marketing, safety, sustainability, and ethics are also discussed in the context of industrial viability and consumer satisfaction.

## 1. Introduction

The nanoscale properties of materials emerge from atomic and molecular configurations yet simultaneously govern the microscopic and macroscopic characteristics of bulk materials. This vital link between different scales highlights the necessity of unified principles for applying nanoscience to design diverse architectures, where molecular building blocks serve as the foundational framework. Using nanoscale building blocks and implementing their assembly, nanoarchitectonics entails the core of processes and assembly procedures allowing the fabrication of nanoscale structures, which are the basis for structures with larger dimensions. Using the principles of nanotechnology, novel molecular structures are built using nanoarchitectonics, which is an architype and core of nano-assembly methods. Initial insights into the subject were discussed by Aono and Ariga [1]; since then, broader perspectives have been gained. Peculiarly, the roots of nanoarchitectonics can also be traced to ancient philosophers [2], and specifically to Aristotle, who advocated for techniques that were closely linked to architectonics that at the time were necessary for construction. In the last century, nanotechnology has received attention following the gaining of understandings of the structure of atoms and molecules and the development of theory. Assembly methods play an important role in this area, where self-assembly at the interfaces [3] and non-perfect self-assembly [2] are some of the main areas of interest. In addition, different applications have been developed and explored where nanoscale phenomena are essential to exploring academic/research and industrial applications across different sizes and scales [4].

Food packaging represents a very important link in the food chain, assuring a safe food supply and delivery, for which protection, preservation, and transportation are needed. Materials [5] constitute an important part of nanoarchitectonics [6], and they are equally important for food packaging. This is because designing novel food packaging approaches relies substantially on new materials. Initially, packaging using plastics appeared very successful and economical but was later reported to be potentially harmful for the health of organisms and humans. Indeed, low degradable plastic has been one of the most often used materials for packaging, which in turn represents the largest market for plastics [7]. And although the cost of plastic production can be as high as EUR 1000 per ton [8] and their separation [9] and labeling [10] is challenging, it is their health risks [11], particularly invisible micro- and nano-plastics, and environmental pollution that necessitate the development of new sustainable approaches and green materials. Various reports are available on the development of novel materials for packaging [12,13,14], but the majority focus on specific materials, where substantial attention was given, not surprisingly, to polysaccharides, which represent an important and growing constituent of green, recyclable, and biodegradable natural products. The general trend of sustainability, recyclability, and green chemistry is distinct in the latest developments. To the best of our knowledge, an analysis of a more general picture of food packaging in the context of nanoarchitectonics, where materials, although they play a very important and even central role, are one of the main subjects of investigation, has not been presented so far.

Here, we discuss a new application area where the principles of nanoarchitectonics are becoming relevant—food packaging. The significance of this field arises from the global emphasis on sustainable practices alongside industrial scalability and economic feasibility. Such analysis would include, besides materials, the type of food to be packaged, protected, transported, environmental factors against which protection is needed, methods of assembly, structure investigation, and societal factors. The link between research and technological development, societal factors, industrial/consumer needs, and preferences in this area are also discussed. Concurrently, there is significant potential to implement knowledge and principles for applied sciences and bio-science engineering to engineer novel materials, sensors, and protection means to ensure that food products are safe, appealing to customers, and economically viable for producers.

## 2. Nanoarchitectonics in the Context of Food Packaging

**Figure 1:**
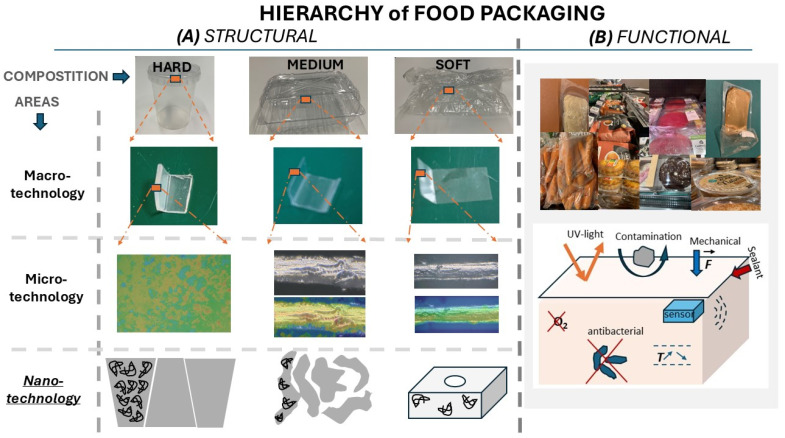
Hierarchy of food packaging. (**A**) Structural composition of food packaging showing its constituents depending on the size and scale. Nanoarchitectonics (underlined in *italic* at the bottom) schematically shows the density of both molecules and package walls as it determines the properties at the micro- (investigated by digital microscopy) and macro- (photographed from food packages available in a supermarket) scales. (**B**) Functional properties nanoarchitectonics identifying protection against bacteria, oxygen, contamination, bacteria, mechanical damage (schematics at the bottom), and food packaging available in a supermarket (top).

Similarly to how an architectonics tree provides connections to the construction of a building [1], Figure 1 shows a hierarchy of food packaging from structural (Figure 1A) and functional (Figure 1B) points of view. Looking at structural building blocks, food packaging can be split into nano-, micro-, and macro-scale structural blocks or areas. In these areas, nanoarchitectonics (at the bottom) determines properties of micro- and macro-building blocks. Looking at the structure of food packaging materials from the composition point of view, it can be noticed that molecules can be packed differently (denser or more disperse), which in turn affects the permeation of aqueous solutions and gasses, leading to harder or softer (images at the top of Figure 1A) packages. Digital microscopy images in Figure 1A depict micro-scale differences between different packages, which can be hard, medium, or soft at the macro-scale. As was reported earlier [1], construction at the nanoscale (nanoarchitectonics) does not always follow the initial design, while in the area of food packaging, this constraint is augmented by very different needs for packaging of different foods.

The functional properties of food packaging show the most important function—protection against various factors (oxidation, light, microbial contamination, temperature, and mechanical actions), Figure 1B.

Further discussion in this review describes different types of food needing packages, Section 3, followed by environmental factors to be protected against (Section 4) and methods of assembly (Section 5). Materials for food packaging (Section 6), administrative and societal factors (Section 7), and conclusions (Section 8) form the rest of this review. Examples of nanoarchitectonics are present in all sections.

## 3. Foods That Are Packaged and Protected: Meat-, Fish-, Dairy-, Plant-, Bakery-, Gelled-, and Beverage-Based Products

In short, almost all types of food need to be packaged and protected, and not only for transportation. Meat is discussed first, followed by other foods.

### 3.1. Meat Protection—Meat Protection Can Be Specific for Different Types of Meat, Where Beef, Poultry, Pork, Lamb, and Processed Meat Can Be Distinguished

Beef. Although new trends in food are developed, fresh beef remains an important food source accounting for over two billion kilograms and over billions in sales annually [15]. Various types of packages exist depending on the necessity of storage for fresh, cooked, or frozen products, due to various environmental factors and conditions that can affect meat. That necessitates the development of new means of allowing the monitoring of the state of sensors for food, meat in particular, and to develop novel materials with the required level of protection.

Indeed, such factors as oxidation, solar radiation, temperature increases, and bacterial and microbiological environments may inflict essential damage to many products, including meat. So, the function of packaging is to protect against these processes and ensure good conditions during storage; here, materials play an important role. Pre-packaging conditions [16], temperature cycles [17], and aging [18] were known long ago to influence the shelf life of meat. This was also reflected in a Food and Drug Administration reports [19,20]. A controlled atmosphere and the offset of other gasses can reduce the oxygen concentration [21], which is one of the most important causes of spoilage [22]. Even if meat products were well packaged, monitoring meat quality represents an important aspect of innovative packaging and, in this regard, gas sensors based on Au containing Ag-SnO_2_/SiO_2_/Si were shown to be effective [23]. Gas sensors, which are necessary for automotive, mining, chemical, mining, and environmental industries, are thus also necessary for the food industry. Similar approaches were described for both meat and chicken products.

The development of novel materials provides innovative solutions to tackle the problems discussed above. Some examples include multi-cross linking and edible hydrogels containing gelatin and k-carrageenan, where dynamic and sufficiently strong cross-linking was performed based on tannic acid with phenolic hydroxyl groups [24]. The optimal performance was found for hydrogel with a concentration of gelatin of 16%. Such materials achieved 73% elongation at break, 72% toughness, over 27% swelling ratio, and Young’s modulus of 50 N. Capability to wash such materials at 40 °C for 30 min represents an essential added value. Cellulose nanocrystals inspired by nanostructured insect wings were also proposed for meat preservation [25]. Hydrochloric acid hydrolysis was used to prepare such films, which showed reinforcement of tensile strength from 74 MPa to over 100 MPa and reduced the water barrier properties from 1.83 g/(m h Pa) to about 1.2 g/(m h Pa). Such materials lowered the log-reduction against *E coli* (0.83) and *Staphylococcus aureus* (0.69) after 5 min of contact time, providing good sustainable alternatives for existing approaches. As is discussed above, silver-containing packaging materials have been identified as effective antibacterial materials [26], where it was shown that the shelf-life of packaged meat was not affected, but meat microbiota was inhibited by adding nanoscale silver coating.

But how is it possible to judge if spoilage is taking place? Different indicators exist for freshness, including color, odor, and general appearance. The color of meat is determined by iron-containing myoglobin, and its changes will lead to the color changes in meat. On the other hand, it may serve as a binding site for biochemical compounds, which would lead to the color, state, and odor/flavor changes [27]. The shelf-life for consumers is first and foremost associated with color and then odor of meat. To maintain its red color, oxygen exposure is needed, while it leads to potentially undesired oxidation of proteins and lipids [28]. Other factors potentially leading to spoilage are bacterial growth and environmental conditions such as temperature, light, etc.

Poultry meat. For poultry, similar protection steps need to be taken as for beef. The modified atmosphere packaging has been long identified to influence the products [29], where optimizing carbon dioxide and nitrogen gasses upon packaging was shown to increase the shelf life from 10 to 14 days. In a more recent study [30], it was shown that carbon dioxide and nitrogen influence the shelf life of poultry. The shelf life was significantly extended for roasted chicken under modified atmosphere packaging, while the influence of other gasses on the shelf life, pH, color, and fat oxidation of chicken was not observed.

Another challenge for poultry is oxidation and exposure to harmful UV light. Assessing lipid peroxide values, thiobarbituric acid, and color variability, it was shown that the incorporation of 30% cellulose nanocrystals into alginate films resulted in enhanced UV barrier effects and 25% decreased oxygen permeability [31]. Light-emitting diode (LED) UV light technology can be used as a practical and economically effective method of disinfection. Monitoring pH, texture, and color following LED-UV exposure was shown to reduce colony forming units (CFU) of different bacteria [32]. Materials continue to play an important role in designing effective packaging solutions. In this regard, plant-based cassia gum edible packaging solutions with added partridge tea extracts exhibited good antioxidant properties for chicken jerky [33].

Pork. Although pork is also a meat, the stringent standard for its good quality often necessitates special approaches. As for other types of meat, lipid and myoglobin oxidation pose challenges in pork [34]. This necessitates the application of controlled atmosphere and vacuum packaging [35], and carbon dioxide was shown to have influence on the spoilage of pork [36]. In addition to the modified atmosphere for packaging, treating pork at high pressure [37] or with organic solvents (acid) [38] is a possible solution. Developing innovative packaging materials and solutions is an important step in providing innovative solutions to some of the above-mentioned challenges [39].

Lamb and other meat and processed products. The development and improvement of methods of storage, as well as being necessary for that research in the area of lamb and other meat products, is oriented to consumer perception [40] which is determined by color and odor, which in turn depend on the preparation methods [41]. To optimize product development, expert panels are brought together [42] to meet the requirements of Bueno, Campo, Cacho, Ferreira, and Escudero [41]. Different analytical techniques are available for assisting the manufacturers to assess consumer assessment [43]. Furthermore, antioxidation [44] and the presence of microbial communities [45] need to be minimized, for which monitoring is of particular importance [46].

The principles affecting the quality of products described above are quite general and applicable to different types of meat [47].

### 3.2. Fish and Seafood

With over 223 million tons of fish, over 185 million tons of aquatic animals, and 37 million tons of algae, the importance of fish in the global food chain is difficult to overestimate. Indeed, it constitutes over 20 kg per capita of worldwide consumption; in 2022, the production of fish and fish-products climbed to a new high [48]. Fish products are valued not only for their contribution to feeding the global population and food security, but also for their valuable nutritional values, particularly omega-3 fatty acids and nutrients. And they provide approximately 15% of global animal protein consumption, with a few countries in Africa and Asia accounting for up to 50% [48]. Although the treatment with temperature, modified atmosphere treatment [49], and UV light [50] for fish is somewhat similar to that for meat, treatment with salt [51] and smoke [52] are more often applicable to fish products, which are favored for their strong antibacterial effects [53].

What makes fish and seafood special in the overall chain is their value for sustainability, more generally, for food packaging. For example, waste streams from crabs and lobsters constitutes 30–40% [54], while that from other fish can constitute up to 20–60% of their weight [55]. But that waste is rich in materials such as chitosan, chitin, alginate, collagen, and gelatin [56]. This represents an important reason for the growth of the consumption of these food sources which needs particular attention regarding packaging due to their usefulness for sustainability.

### 3.3. Dairy Products

Packaging milk and dairy products is different from meat, since most consumers have experience with milk spoilage. The storage conditions appear to be essential for maintaining milk in good condition, but the materials used for packaging also play a very important role. In a recent study [57], the properties of milk packaged in paper-board cartons, low-density polyethylene (LDPE), high-density polyethylene (HDPE), polyethylene terephthalate (PET), and glass were compared. For this purpose, equal volumes of milk (~280 mL) pasteurized at high temperature (77 °C for 25 s) were poured into packages composed of the above-mentioned materials and were stored at 4 °C in a dark environment. The sampling was performed on days 0, 5, 10, and 15, and it was found by consumers and a trained panel that milk packaged in paper-board cartons and LDPE had the highest intensities of off-flavors, presumably due to permeability and migration. It was also found in that study that milk packaged in HDPE, PET, and glass containers had no detectable sensory differences by day 10 of storage. Temperature, oxygen scavengers, and antimicrobial control are very important factors in the preservation of dairy products [58]. As can be linked with the above examples, the application of advanced analytical techniques, including gas-chromatography-mass spectrometry, can also be very useful for the identification of migrating chemicals from polyethylene-based multilayer packaging [59].

Cheese packaging is another application area in this section, where the emphasis lies on recyclable packaging solutions. Four different multilayered packages were studied composed of: (1) polyvinylidene (PVDC) and polyethylene (PE), used in cheese packaging by Amcor Plc (Victoria, Australia) and referred to as MultiVAC; (2) layered materials consisting of PE and polyamide (PA), used by GM Grafica s.r.l. (Cerbara, Italy) and referred to as MultiMAP; and (3) polypropylene (PP) with high-barrier properties (HB-PP/cast (C)-PP (MonoMAP1) as well as biaxially oriented BO-PP/C-PP (MonoMAP2) and supplied by Poplast s.r.l. (Castel San Giovanni, Italy). The latter packages had 94% recyclability (according to CYGLOS-HTP) and low barrier properties O_2_: 13–20 cm^3^ (STP)/(m_2_ × 24 h) and CO_2_: 60–90 cm^3^ (STP)/(m_2_ × 24 h), both at 0% relative humidity. The shelf-life assessed by a rancidity indicator showed that the application of 1-layer packaging material (MonoMAP1) significantly reduced the shelf-life compared to that of multi-layer packaging [60]. A brief summary of different materials is presented in Table 1.

However, the development of novel packaging solutions and materials with good sustainability, effective oxygen scavengers, and multilayer preservation is very important for different dairy products.

### 3.4. Plant-Based Products: Fruits, Vegetables, and Shell-Based Products

While the protective requirements for fruits and vegetables differ markedly from those for meat or dairy products, mitigating microbial contamination remains equally vital in both areas [61,62]. In this regard, the application of different processing methods, such as high pressure, ultrasound, pulsed electric fields [63], and UV-light [64], was studied. Novel developments include edible coatings with advanced antibacterial, antioxidant, antifogging, washable, and strong mechanical properties [65]. But the quality of fruits and vegetables has to be carefully monitored and considered while developing such coatings [66]. It should be noted that some fruits and vegetables already have a protective coating, for example nuts, so packaging is quite relevant for transportation, but recyclability remains an important aspect [67].

### 3.5. Other Products: Gelled, Cans, Bread and Patisserie, Beverages and Drinks, Etc.

Various other products exist where special conditions need to be maintained. For example, for gelled products, the microstructure of packaging needs to be studied [68] to ensure necessary permeability.

Many other products exist where packaging is also used. For example, packaging for bread and patisserie products has been quite different compared to other food products, because freshness can be well preserved using paper- and cellulose-based packages.

The needs and challenges in beverage and drinks packaging differ substantially from those in other areas. In this area, glass or aluminum containers are used in addition to Tetra Paks and PET bottles, as shown in Table 1. Different types of cans are also broadly used in packaging [69], where the aims are also directed at recyclability and safety.

### 3.6. Nanostructure of Food

Extensive detailing of the structure of various foods is outside of the scope of this work, but it is worth mentioning that nanostructures in food have received attention due to the importance of security and safety [70]. An important application area here is extraction [71,72], where the methods often used for the synthesis of new materials [73]—microwave, ultrasound, mechanical extraction, enzymatic reactions—are applied for the extraction of proteins, lipids, and carbohydrates [74] from foods and side-streams [75]. In addition, analytical and microscopy tools are important for the investigation of food nanostructures, where, for example, cryo-Electron and Raman microscopy techniques were used to investigate hydrogen bond formation while probing the interaction between lecithin and fruit wax for the formation of oleogels [76]. Furthermore, Raman microscopy can be used to investigate the degradation of molecules [77]. It is also impossible not to mention the latest trends in this area, where advanced machine and deep learning algorithms are applied to investigate the structures of materials, for example, predicting the polymorph phases of calcium carbonate [78] and their link with specific processes, such as lipid oxidation, with Raman microscopy [76]. Furthermore, fluorescence and Raman microscopy were shown to be of particular interest in monitoring protein aggregate formation [79] and microalgal lipid body formation [80]. In addition, such advanced analytical tools as X-ray scattering, cryo-electron microscopy, differential scanning calorimetry, and microscopy, including polarization microscopy, are of crucial importance in assessing relevant processes, for example, crystallization [81].

## 4. What Environmental Threats to Protect Food Against

Physical, chemical, or metabolic changes in food products during storage and transportation are unconducive to maintaining the original quality of foods. Several influence factors, including oxygen, bacteria, light, temperature and mechanical injury, contribute to food spoilage.

### 4.1. Oxygen

Oxygen plays a central role in food spoilage by enabling aerobic microbial growth and driving oxidative chemical reactions [82]. Microorganisms such as *Pseudomonas* sp. metabolize lipids and proteins in the presence of oxygen, producing off-flavors and odors [83]. Non-microbial spoilage involves the oxidation of unsaturated fats, where oxygen reacts with fatty acids (e.g., linoleic acid) to form unstable hydroperoxides [84]. These compounds further decompose into aldehydes and ketones, responsible for rancidity in oils and fatty foods. Enzymatic browning, mediated by polyphenol oxidase, oxidizes phenolic compounds in fruits and vegetables (e.g., apples, potatoes) into dark pigments [85]. Even in modified atmosphere packaging (MAP), residual dissolved oxygen in food matrices can sustain oxidation, necessitating oxygen scavengers or antioxidant additives to maintain quality [86,87].

### 4.2. Bacteria

Spoilage bacteria, such as *Pseudomonas* and *Shewanella* [88], break down proteins and lipids, producing volatile sulfur compounds and amines that cause unpleasant odors (e.g., “fishy” smells in seafood). Pathogens like *Listeria monocytogenes* and *Salmonella* thrive and are microorganisms that proliferate in various food matrices, posing significant public health risks through the onset of listeriosis and salmonellosis, respectively [88,89]. Biofilms, structured communities of bacteria embedded in protective extracellular matrices, form on food surfaces, packages, and equipment, resisting sanitation efforts. The complex structure of biofilms and quorum sensing enhance bacterial survival, making them resistant to antimicrobial treatments [90]. Factors such as pH, water activity, and nutrient availability dictate microbial growth rates. For example, lactic acid bacteria dominate in low-pH environments (e.g., fermented dairy), converting sugars into lactic acid and extending shelf life through natural acidification [83,91].

### 4.3. Light

Light exposure, particularly ultraviolet (UV) and visible wavelengths, accelerates food degradation through photochemical reactions [92,93]. UV light damages microbial DNA, reducing surface contamination, but also degrades photosensitive nutrients like riboflavin and chlorophyll [94,95]. In lipids, light initiates oxidation, forming free radicals that propagate chain reactions, leading to rancidity [96]. Transparent packaging, while visually appealing, allows light to penetrate and degrade vitamins and pigments (e.g., anthocyanins in berries) [97]. Advanced packaging materials incorporate light-blocking nanoparticles (e.g., titanium dioxide) or use amber-colored films to filter harmful wavelengths while maintaining product visibility [98,99].

### 4.4. Temperature

Temperature fluctuations during storage and transport are critical determinants of food quality [100]. Elevated temperatures accelerate enzymatic reactions (e.g., browning in cut fruits) and microbial growth, while freezing can damage cellular structures in fresh produce, leading to texture loss upon thawing. Psychrotrophic bacteria, such as *Listeria*, grow slowly even at refrigeration temperatures, necessitating strict cold chain management [91,101]. Temperature abuse during distribution (e.g., prolonged exposure to heat) can also induce protein denaturation in meats and dairy, altering texture and flavor [102]. Innovations like phase-change materials in packaging help buffer against temperature variations, maintaining optimal conditions for perishable goods [103].

### 4.5. Mechanical Injury

Physical damage during harvesting, handling, or transport compromises food integrity. Bruising or crushing plant tissues releases enzymes like polyphenol oxidase and pectinase, which catalyze browning and soften textures [104]. Mechanical injury also creates entry points for microbial invasion, accelerating spoilage. For example, bruised apples exhibit increased ethylene production, hastening ripening, and senescence [105]. Protective packaging designs, such as shock-absorbing foam or rigid containers, minimize mechanical stress, while automated sorting systems reduce handling-induced damage in fresh produce [106,107].

### 4.6. The Role of Nanoarchitectonics in Protection Against Environmental Factors

Nanoarchitectonics, with its extensive capability of assembly at the nanoscale, plays an important role in assembly methods which will be discussed in the next section. Here, we would like to mention that environmental threats can be offset by (a) monitoring the effects of environmental threats and (b) providing effective and necessary protection by providing appropriate packaging solutions.

## 5. What Methods of Assembly, Structures, and Property Investigation Are Available

Nanomaterials for food packaging are engineered via bottom-up (e.g., layer-by-layer assembly [108], sol–gel synthesis [109]) or top-down (e.g., electrospraying [110]) strategies to create nanocomposites, nanocoatings, or nanofibrous networks with tailored barrier and antimicrobial functionalities (Figure 1B). Hierarchical architectures, such as metal–organic frameworks (MOFs) [111] or core–shell nanoparticles [112], optimize gas adsorption and the controlled release of preservatives. Meanwhile, for the property investigation, structural characterization employs SEM/TEM for nanoscale morphology, and FTIR/XRD analyze molecular interactions [113]. Mechanical and barrier properties are tested via ASTM standards [114], and antimicrobial efficacy is validated through microbiological assays [115]. These integrated approaches ensure that nanoarchitected materials effectively meet complex preservation requirements while simultaneously addressing critical safety and sustainability considerations.

### 5.1. Active Packaging Materials and Molds

Active packaging integrated with nanoarchitectonics represents a cutting-edge approach to mitigate microbial contamination, which is a pervasive challenge in food spoilage. These systems employ nanomaterials with inherent antifungal properties, such as silver nanoparticles (Ag NPs) [116,117], zinc oxide nanoparticles (ZnO NPs) [118], and chitosan-based nanocomposites [119], which disrupt microbial growth through targeted mechanisms. For instance, Ag NPs destabilize fungal cell membranes by binding to thiol groups in proteins and inhibiting ATP synthesis, while ZnO NPs generate reactive oxygen species (ROS) that degrade cellular components of microbes like *Aspergillus* and *Penicillium* species [120,121]. The integration of these nanomaterials into packaging matrices—via methods such as solvent casting, electrospinning, or layer-by-layer assembly—enables the controlled release of antifungal agents, ensuring sustained protection throughout storage [122,123]. Nanocomposite films incorporating montmorillonite clay or cellulose nanocrystals further enhance barrier properties, reducing moisture uptake and oxygen permeation, both of which are critical for microbial proliferation [124]. The uniform dispersion of nanoparticles within polymer matrices was generally revealed by scanning electron microscopy (SEM) and atomic force microscopy (AFM), and the antifungal assays (e.g., agar diffusion tests) quantify their efficacy against common microbial spoilage [87,125].

### 5.2. Barrier Permeations

Barrier permeation properties are pivotal factor for food packaging, as they govern the transmission of gasses (e.g., O_2_, CO_2_), moisture, and volatile organic compounds (VOCs) through packaging materials. Uncontrolled permeation accelerates oxidative rancidity, microbial growth, and flavor loss, significantly reducing shelf life [126]. Nanoarchitectonics addresses these challenges by engineering advanced barrier systems through the integration of nanofillers—such as montmorillonite clay, graphene oxide, or cellulose nanocrystals—into polymer matrices. These nanofillers create tortuous diffusion pathways, drastically impeding the movement of gas and water molecules due to their high aspect ratio and interfacial interactions with the polymer. For example, hydrophobic nanofibers (e.g., alkyl ketene dimer spray-deposited on nanocellulose) enhance moisture resistance in biodegradable films, preventing water vapor ingress that promotes mold growth [127]. Beyond passive barriers, active nanocomposites can dynamically respond to environmental stimulus. For instance, pH-sensitive nano-coatings may selectively release antimicrobial agents under spoilage conditions, while smart films embedded with nano-sensors monitor and adjust permeability in real time [122]. The quantification of barrier efficacy and the correlation of nanomaterial structure with performance are commonly characterized by oxygen transmission rate (OTR) analysis, water vapor permeability (WVP) testing, and synchrotron-based X-ray scattering [128,129]. By optimizing these properties, nanoarchitectonics enables thinner, lighter, and more sustainable packaging solutions without compromising protective functionality, aligning with global efforts to reduce plastic waste [129].

### 5.3. Plasma Treatment

**Figure 2:**
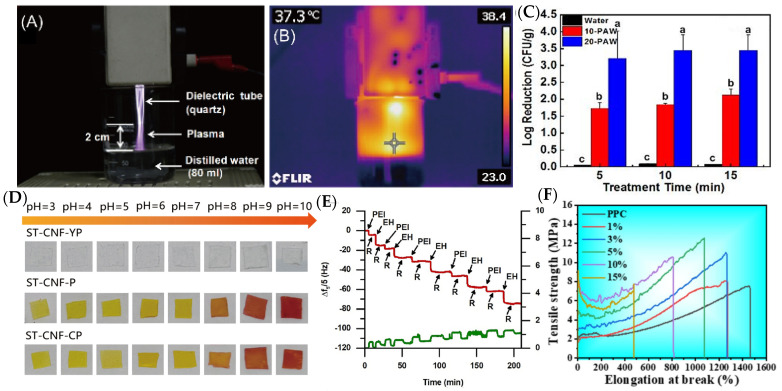
(**A**) A photograph of PAW generation. (**B**) The thermographic measurement of plasma near the water surface during PAW generation.z (**C**) PAW inactivation efficacy against *S. aureus* inoculated on strawberries with 4-day storage, columns with different letters indicate a significant difference (*p* < 0.05) (adapted from [132] with permission from Elsevier). (**D**) Images of colorimetric response of curcumin solution and films in buffers with different pH values: films for monitoring pork freshness (adapted from [119] with permission from Elsevier). (**E**) Changes in oscillating frequency (red or blue line, left axis), from the 5th overtone from the QCM-D measurements, and energy dissipation (green line, right axis) as a function of time, recorded during the deposition of five bilayers for the PEI/EH system at pH 8 (adapted from [108]). (**F**) Longitudinal tensile strength of film (adapted from [134] with permission from Elsevier).

Plasma treatment, a non-thermal surface-modification technique, has emerged as a versatile method to enhance the functionality of packaging materials and food surface modifications for longer preservation [130]. By generating reactive oxygen and nitrogen species (RONS), UV photons, and charged particles, cold plasma modifies surface chemistry and topography at the nanoscale, improving adhesion, wettability, and antimicrobial properties [131]. For instance, plasma-activated water (PAW) or direct plasma exposure can decontaminate food surfaces by disrupting microbial cell membranes and oxidizing vital biomolecules (Figure 2A,B), effectively reducing pathogen loads without compromising food quality [132]. In packaging applications, plasma treatment is used to functionalize polymer surfaces, enabling the uniform deposition of nanostructured coatings (e.g., silicon dioxide or titanium dioxide nanoparticles) that enhance barrier properties against oxygen and moisture [133]. Furthermore, plasma-assisted grafting of antimicrobial agents onto packaging films produces active surfaces capable of effectively suppressing spoilage microorganisms. As an eco-friendly, residue-free method, this innovation represents a promising advance in sustainable food preservation, aligning with modern demands for safer and greener solutions.

### 5.4. Sensors for Intelligent Packaging and Monitoring

Intelligent packaging systems equipped with nanoarchitectonics-based sensors are revolutionizing food preservation by enabling real-time monitoring of freshness, safety, and storage conditions [91]. These sensors leverage nanomaterials such as carbon nanotubes, graphene, and metal–organic frameworks (MOFs) to detect critical biomarkers, including gas emissions (e.g., CO_2_, ethylene, ammonia), pH changes, and microbial metabolites, which signal spoilage or contamination [97]. For instance, carbon nanotube-based gas sensors exhibit high sensitivity to ethylene, a ripening hormone in fruits, allowing precise tracking of post-harvest quality [101,135]. Similarly, colorimetric nano-sensors functionalized with gold or silver nanoparticles undergo visible color transitions in response to spoilage-induced pH shifts or pathogen presence, providing intuitive visual alerts to consumers [136].

Nanofabrication techniques, such as inkjet printing and electrospinning, enable the integration of these sensors into flexible packaging substrates without compromising structural integrity [137]. Meanwhile, wireless communication technologies, including radio-frequency identification (RFID) tags and near-field communication (NFC) systems, further enhance functionality by transmitting sensor data to smartphones or supply chain management platforms, facilitating dynamic shelf-life predictions and traceability [136]. Despite their potential, challenges remain in scalability, cost-effectiveness, and regulatory compliance. Emerging research is prioritizing biodegradable nano-sensors crafted from renewable biopolymers like cellulose or chitosan, offering dual advantages: they resolve sustainability challenges while delivering uncompromised performance (Figure 2D) [122]. By bridging nanotechnology with Internet of Things (IoT) capabilities, intelligent packaging systems promise to minimize food waste, optimize supply chains, and empower consumers with actionable quality insights.

However, current nano-sensors face challenges in sensitivity (interference from environmental variables), stability (degradation under thermal/mechanical stress), and scalability (high-cost, low-yield fabrication). Regulatory uncertainties regarding nanoparticle migration and biocompatibility, coupled with hurdles in integration into conventional packaging systems, further limit commercialization. Addressing these requires harmonized advances in material robustness, scalable manufacturing, and standardized safety protocols to transition lab innovations into practical, reliable solutions.

### 5.5. Thermal Treatment

During synthesis, technologies such as sol–gel processing and electro-spraying require precise thermal control to stabilize nanoparticle formation and prevent degradation of heat-sensitive bioactive agents (e.g., antioxidants) [138]. Furthermore, thermo-responsive polymers, such as poly(N-isopropylacrylamide), enable smart packaging systems that modulate permeability or release antimicrobials in response to temperature fluctuations during storage or transport [139]. Typically, structural stability under thermal stress is evaluated using thermogravimetric analysis (TGA) and differential scanning calorimetry (DSC) to identify decomposition thresholds and phase transitions— essential parameters for ensuring the reliability of applications such as microwave-safe packaging [140]. Barrier properties, including oxygen transmission rate (OTR) and water vapor permeability (WVP), are temperature-sensitive; accelerated aging tests simulate real-world conditions to validate performance across cold chain or high-temperature environments [102]. Such thermal optimization ensures packaging integrity and active functionality across diverse preservation scenarios.

### 5.6. Light Treatment: Ultraviolet (UV), Visible, Pulsed, and Radiation

Light-based interventions, including ultraviolet (UV), visible light, pulsed light, and ionizing radiation, are increasingly integrated with nanoarchitectonics systems to enhance food preservation efficacy. Nanocomposite films embedded with UV-blocking nanoparticles (e.g., ZnO or TiO_2_) protect photosensitive foods (e.g., lipids, vitamins) from oxidative degradation while permitting selective UV-C sterilization [101,118]. Visible light-activated photocatalysts, such as TiO_2_ or carbon quantum dots, generate reactive oxygen species (ROS) under illumination, offering continuous antimicrobial action without residue [97]. Pulsed light (broad-spectrum, high-intensity flashes) inactivates surface microbes through photothermal and photochemical effects, with nano-silver or graphene oxide enhancing absorption efficiency [141,142]. Ionizing radiation (gamma, X-ray, or electron beam) penetrates bulk packaging to sterilize food by disrupting cellular structures; nanoparticle-enhanced radiosensitizers (e.g., gold nanoparticles) amplify radiation’s microbial lethality while minimizing dose requirements [143]. Nano-structured light-responsive systems offer energy-efficient, targeted regulation of spoilage pathways, ensuring robust preservation of food safety and quality.

### 5.7. Assembly Methods and Structure: Microfluidics and Janus Structures

The nanoarchitectonic design of advanced food packaging materials relies on precise assembly methods to engineer hierarchical structures with tailored functionalities. Microfluidics has emerged as a versatile platform for constructing nanostructured packaging systems, enabling controlled fluid dynamics at the microscale to produce monodisperse emulsions, particles, or fibers [144]. This technique facilitates the encapsulation of active agents (e.g., antimicrobials, antioxidants) within polymer matrices, optimizing their controlled release and spatial distribution [145]. Janus structures, characterized by spatially segregated chemical or physical domains within a single entity, offer dual functionalities critical for active and intelligent packaging. These asymmetrical architectures, often fabricated via interfacial polymerization or phase-separation techniques, enable synergistic effects such as simultaneous barrier enhancement and stimuli-responsive behavior [146]. A notable example includes amphiphilic Janus particles, where hydrophobic and hydrophilic domains coexist, improving compatibility with polymer matrices while enabling the targeted release of bioactive compounds [147,148,149]. Such structures are particularly advantageous in packaging systems requiring pH- or temperature-triggered antimicrobial action.

### 5.8. Enhancement of Mechanical Properties Needed for Transportation

Nanoarchitectonics enhances the mechanical robustness of food packaging by leveraging nanoscale reinforcements and structural optimization to achieve high strength-to-weight ratios, flexibility, and durability, ensuring resilience against physical stresses like impact, compression, vibration, and temperature fluctuations. A prominent approach involves the integration of nanofillers, such as cellulose nanocrystals (CNCs), graphene oxide (GO), or montmorillonite (MMT) clay, into polymer matrices [150]. These nanofillers act as stress-transfer agents, enhancing tensile strength, Young’s modulus, and fracture resistance by restricting polymer chain mobility and dissipating energy under load [151]. For example, adding CNC into k-carrageenan biopolymer matrix, via solvent casting method, resulted in nanocomposite films with largely improved tensile strength and toughness parameters [150]. Similarly, a 150% improvement of tensile strength of the graphene/PVA composites is obtained at low graphene loading [152].

Hierarchical structuring further optimizes mechanical performance. Layer-by-layer (LbL) assembly or 3D-printed lattice architectures enable precise control over mechanical properties (Figure 2E,F), mimicking natural composites like nacre or bamboo [108,134]. Such biomimetic designs combine rigid and elastic phases, achieving synergistic toughness and crack deflection. Scalability remains a key consideration. Melt-blending techniques compatible with industrial extrusion processes ensure uniform dispersion of nanofillers, while solvent-free methods align with sustainability goals. By balancing mechanical resilience with lightweight design, these innovations reduce material waste and energy consumption in logistics, supporting the transition toward circular economy principles in food packaging.

### 5.9. Sealants

Bioinspired adhesive systems mimic the hierarchical structures of gecko footpads or mussel byssal threads, achieving reversible adhesion through nanoscale patterning or dopamine-based coatings [153]. Additionally, pH-sensitive hydrogels functionalized via signaling cascade amplification can autonomously adjust their swelling behavior, forming self-tightening seals under humid conditions to counteract package expansion during transportation [154]. These systems are particularly advantageous for resealable packaging, reducing food waste by enabling repeated access without compromising seal integrity.

Layer-by-layer (LbL) assembly further optimizes sealant performance by engineering multilayered architectures with spatially graded properties. For example, alternating layers of cationic chitosan and anionic polyacrylic acid, interspersed with silver nanoparticles, create antimicrobial seals with dual functionality: robust adhesion and pathogen inhibition at the package interface [155]. Advanced deposition techniques, such as electro-spraying or plasma-enhanced chemical vapor deposition, allow precise control over sealant thickness (nanometer to micrometer scale) and uniformity, minimizing material usage while maximizing performance [156]. Computational modeling of stress distribution at seal interfaces guides the design of failure-resistant geometries, enhancing durability under mechanical or thermal stress [157]. Furthermore, water-based nano-sealants and UV-curable formulations reduce reliance on volatile organic compounds (VOCs), aligning with green packaging initiatives [158].

### 5.10. Analytical Techniques for Property Investigation and Characterization: Imaging, Spectroscopy, Analytics

The precise evaluation of nano-architecture food packaging materials relies on advanced analytical tools to probe structural, mechanical, and functional properties. Imaging techniques, such as scanning/transmission electron microscopy (SEM/TEM) and atomic force microscopy (AFM), resolve nanoscale morphology, particle distribution, and interfacial interactions within composites [159]. Spectroscopic methods like Fourier-transform infrared (FTIR) and Raman spectroscopy elucidate chemical bonding and molecular interactions, while X-ray diffraction (XRD) quantifies crystallinity and phase composition in nanocomposites [99,117]. Surface-sensitive analytics, including X-ray photoelectron spectroscopy (XPS) and time-of-flight secondary ion mass spectrometry (ToF-SIMS), map elemental composition and degradation pathways at material interfaces [160]. Antimicrobial efficacy is quantified via disk diffusion, minimum inhibitory concentration (MIC), and confocal laser scanning microscopy (CLSM) to visualize biofilm inhibition [119]. Release kinetics of active agents (e.g., antioxidants, antimicrobials) are monitored using high-performance liquid chromatography (HPLC) or UV-vis spectroscopy, coupled with mathematical modeling (e.g., Fickian diffusion) [128,161]. Emerging techniques, such as in situ TEM and hyperspectral imaging, enable real-time analysis of structural dynamics and contaminant detection [162]. These multimodal approaches ensure comprehensive validation of nanoarchitecture systems, bridging laboratory insights into real-world food preservation performance.

### 5.11. Nanoarchitectonics and Assembly Methods and Their Application for Food Packaging

Assembly methods can be viewed as static and dynamic; while static methods rely on equilibrium interaction, dynamic assembly methods can be associated with the application of different stimuli [163]. Even synthesis can be carried out in a dynamic environment [164]. For optimization of assembly, advanced analytical tools can be used for investigating the interaction of, for example, particles and surfaces [165]. Assembly methods for materials [3], among which are layered structures [166], were long-ago considered to be advantageous for different applications. Among these assemblies, layer-by-layer (LbL) assembly [167,168] has been particularly attractive due to its versatility and availability of different stimuli for controlling their properties [169] providing means to enable applications in different areas [170]. For food packaging, different solutions using LbL films have also been offered [171,172,173]. For instance, chitosan (positively charged) and pectin (negatively charged) multilayers embedded with Ag NPs or essential oils (e.g., thymol) exhibited pH-triggered release, targeting spoilage microbes at specific storage conditions, and did not hinder the gas exchange of muskmelon or result in the accumulation of volatile odors [123,174].

## 6. Materials in Packaging: Key Components of Developing Innovative, Sustainable/Recyclable, and Safe Packaging Approaches

Finally, the development new materials and structures, particularly using nanoarchitectonics approaches, represent the corner-stone building blocks of efficient packaging. This is particularly relevant because plastic has so far been used very widely in many industries [7], including packaging, and some of these materials can cause problems in the environment.

### 6.1. From Plastic-Based, to Bioplastic-Based, to Biodegradable Materials

**Figure 3:**
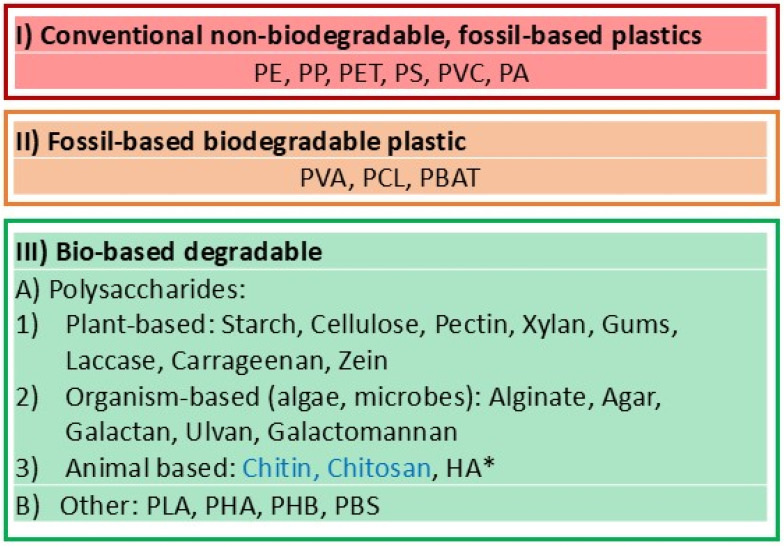
Classification of plastic- and biopolymer-based materials used for packaging. (I) Polyethylene (PE), polypropylene (PP), polyethylene terephthalate (PET), polystyrene (PS), and polyvinyl chloride (PVC). (II) Polybutylene succinate (PBS), polyvinyl alcohol (PVA), polycaprolactone (PCL), and polybutylene adipate co-terephthalate (PBAT). (III) Polylactic acid (PLA), polyhydroxyalkanoates (PHA), polyhydroxybutyrate (PHB), and hyaluronic acid (HA). *—can also be synthesized in laboratory conditions.

The development of novel materials for sustainable, biodegradable, and recyclable food packaging represents one of the most essential goals of modern development. Figure 3 shows a table highlighting (I) conventionally used non-biodegradable and fossil-based plastic packaging materials; (II) fossil-based plastic solutions improved by possibility of biodegradation; and (III) bio-based biodegradable materials, which can be either synthesized or extracted from different sources (plants, organisms, animals).

(I) Conventional polymers for plastic were some of the first.

The most common plastics used in packaging plastics, which are broadly used materials, include: polyethylene (PE), polypropylene (PP), polyethylene terephthalate (PET), polystyrene (PS), and polyvinyl chloride (PVC), which constitute just over 40% of all non-fiber plastics [175]. They were indeed the most often used materials in packaging until safety concerns from packaging solutions emerged. That is why in Figure 3, this class of materials is highlighted in red, because greener solutions are sought in continuing research and development steps.

(II) Fossil-based biodegradable plastic.

Fossil-based biodegradable solutions seemed to be a good solution to address biodegradability, which is why they are highlighted in orange in Figure 3. But the contemporary novel biodegradable biopolymers described in the next section provide very promising new materials.

(III) Bio-based degradable.

Biopolymers with biodegradable functionality represent a large and growing class of materials designated to be used also in packaging. Different polymers and biopolymers for plastic and bio-plastic exist [176] and we analyze two major types: (A) polysaccharides and (B) other materials. From different polymers, PCL can be highlighted as a semicrystalline polymer with a low glass transition temperature and low melting point; it is valued due to its effective blending capability. PBAT is attractive due to its good biocompatibility, high flexibility, and excellent processibility.

(A) Polysaccharides.

Polysaccharides represent an important class of biomolecules where a long polysaccharide chain is composed of smaller monosaccharide units and can be sub-divided according to the source they were produced from: plants, animals, or organisms (microbes, algae) [177]. Some further examples of developing sustainable packaging solutions are presented based on a selection of polysaccharides.

Plant extracts have been shown to be an attractive solution for packaging [178], where control over physical, mechanical, barrier, antioxidant, and antimicrobial properties can be obtained [179]. For example, pectin bioactive composites have been reported to have antibacterial properties [180].

Starches and thermoplastic starches have been modified by ionic liquids [181] as well as reinforcements, modifications, and polymers [182] to improve hydroscopic properties and control permeability. Cellulose-, paper-, and wood-based materials are excellent in terms of sustainability (Forest Stewardship Council™ (FSC™)).

Laccase is a multi-copper oxidase enzyme which promotes the cross-linking of matrix macromolecules, and which is used in food packaging due to its potential for facilitating sustainability and safety. Zein, a protein extracted from corn, is an abundant, amphiphilic protein with good gas barrier properties [183]. Similarly to other types of packaging materials, zein was modified with polyphenolic acids (gallic acid, p-hydroxy benzoic acid, ferulic acid) and flavonoids (catechin, flavone, quercetin) to enhance mechanical properties relevant for packaging [184]. Another interesting material for packaging is carrageenan [185].

Alginate, a naturally occurring biodegradable polysaccharide, is considered to be a promising material for packaging due to its recyclability and versatility [186]. In addition to alginate, agar [187] and gelatin [188] have also been shown to be promising materials.

Chitosan, an abundant biopolymer produced from chitin (by deacetylation), with its excellent antibacterial properties, has long been under the radar of food packaging applications [189], and as it was reported for other packaging solutions, modifications of chitosan are also beneficial for packaging.

Furthermore, industrially relevant enzymes, for example, laccase have also been shown as promising agents for food packaging applications [190].

(B) Other biodegradable polymers: PLA, PHA, PHB, PBS.

PLA, PHA, PHB, and PBS are biodegradable materials displaying high promise as replacements for conventional non-biodegradable plastics. PLA is a biopolymer synthesized by ring-opening polymerization, which is broadly used in food packaging applications due to its mechanical and barrier properties [191]. Often, and similarly as it was for other materials, designing hybrid structures using PLA is gaining attention due to the possibility of adding beneficial properties by incorporating additives. For example, PLA has been modified with glass microspheres [192], curcumin [193], polymers and oils [194], starch nanocrystals [195], algae [196], lignin [197], oils [198], and green tea extracts [199]. Typically, enhanced mechanical, antioxidative, barrier, optical properties can be obtained by fabricating such composite materials. PBS is a biodegradable, according to ISO EN13432 standard, aliphatic polyester with remarkable thermal resistance and heat deflection [200]. Due to its rather subprime mechanical properties, it is often mixed with other polymers, for example, PLA. PBS is an interesting material used for multiple purposes where a balance between mechanical strength and thermal resistance is needed.

### 6.2. Edible Materials as a Developing Trend in Packaging

As was shown in different sections of this review, edible coatings and materials have been shown to be promising agents for packaging. Here, their versatility and relevance to sustainability can be further underlined [201].

### 6.3. Hybrid/Composite Materials and Methods of Their Assembly

**Figure 4:**
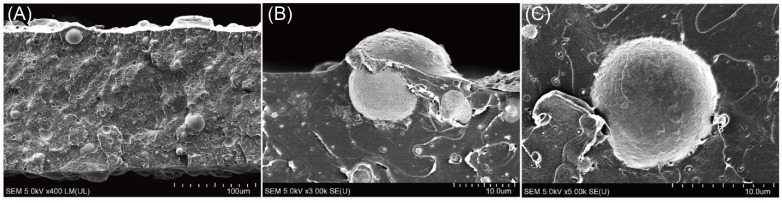
SEM cross-sectional images of glass microbeads in PLA films. (**A**) The distribution of glass microbeads in PLA; (**B**) a closer look and cone structure; (**C**) adhesion to the surface. Reproduced from [192] with permission Elsevier.

Hybrid and composite materials comprise different but complementary components, the classification of which was given for inorganic–in-organic structures [202] as well as organic–in-inorganic assemblies [203]. Their essential advantage their use of complementary properties, identified as ying-and-yang in the above classifications. Nanoarchitectonics of composite materials has been recently highlighted [204], where such complementarity was underlined. Figure 4 shows an example of the incorporation of glass beads into PLA film, where enhanced barrier- and optical-properties have been obtained.

**Figure 5:**
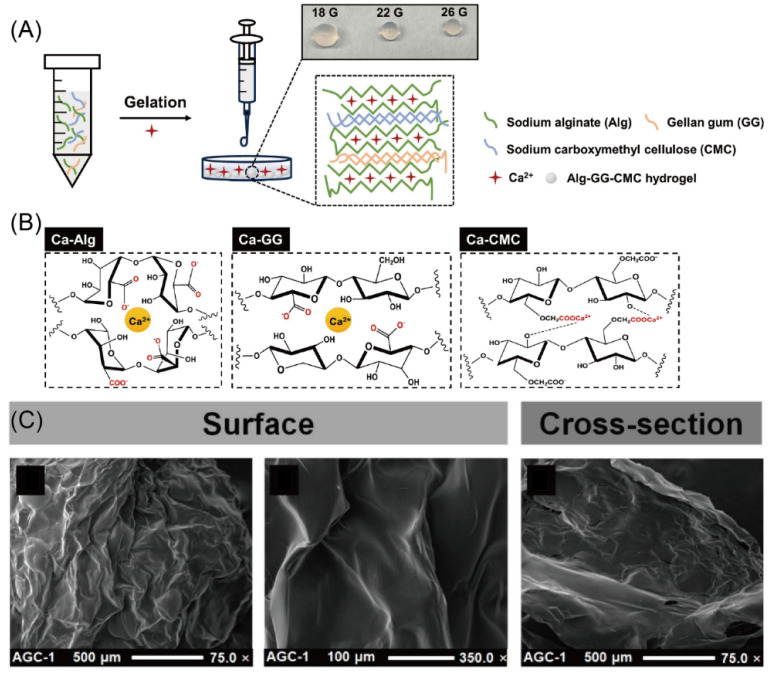
(**A**) Schematic diagram of the preparation process of an alginate–gellan gum–carboxymethyl cellulose (Alg-GG-CMC) hydrogel, where (**B**) molecular and ionic interaction are shown between Alg, GG, CMC, and Ca^2+^ ions, respectively. (**C**) Cryo-SEM images of the surface (reproduced from [214] with permission Elsevier).

Designing new hybrid and composite coatings represents a growing trend [205]. In this regard, Figure 5 shows an example of the fabrication of hybrid material based on different hydrogels, where the flexibility of assembly and advantageous properties highlight their importance not only as an interesting material, but also as potential candidate for functionalizing with antioxidative biomolecules [206].

### 6.4. Nanoarchitectonics and the Role of Materials for Food Packaging

Nanoarchitectonics, which organize nanoscale components into functional hierarchical structures [170,207], directly governs the physicochemical and biological properties of food packaging materials (Figure 1). By engineering interactions between molecules, polymers, nanoparticles, and bioactive agents at molecular to microscale levels, nanoarchitectonics enables precise control over barrier efficiency (e.g., oxygen, moisture), antimicrobial activity, mechanical robustness, and stimuli responsiveness [208]. For instance, aligned clay nanoplatelets in polymer matrices reduce gas permeability by creating tortuous diffusion paths [209], while Ag NPs spatially distributed in coatings provide contact-based microbial inhibition [210]. Such structural precision optimizes performance while minimizing material usage, aligning with sustainability goals. The integration of nanoarchitectonics based on advanced assembly techniques enables multifunctional, application-specific packaging systems. For instance, surface-grafted polymer brushes (e.g., poly(2-vynil pyridine) and poly(3-[dimethyl-[2-(2-methylprop-2-enoyloxy)ethyl]azaniumyl]propane-1-sulfonate)) create dense, hydrated layers that repel microbial adhesion while allowing the selective permeation of gasses [211,212,213]. Their “stealth” properties reduce biofilm formation on meat and dairy packaging surfaces.

The temperature sensitivity of brushes [215] provides an additional mechanism for controlling their properties. Aligned or core–shell nanofibers produced via electrospinning encapsulate antioxidants (e.g., α-tocopherol) or antimicrobial agents (e.g., chlorogenic acid), providing sustained release kinetics [216]. These structures enhance preservation in fresh produce by maintaining high surface-area-to-volume ratios for active compound delivery. Through rational nanoarchitectonics design of functional materials at the nanoscale, next-generation food packaging exhibits multifunctional capabilities, such as self-cleaning surfaces, real-time freshness monitoring, and adaptive barrier responsiveness, among others.

## 7. Administrative and Societal Factors: Safety, Ethics, Sustainability, and Marketing as Key Driving Forces in Food Packaging

**Figure 6:**
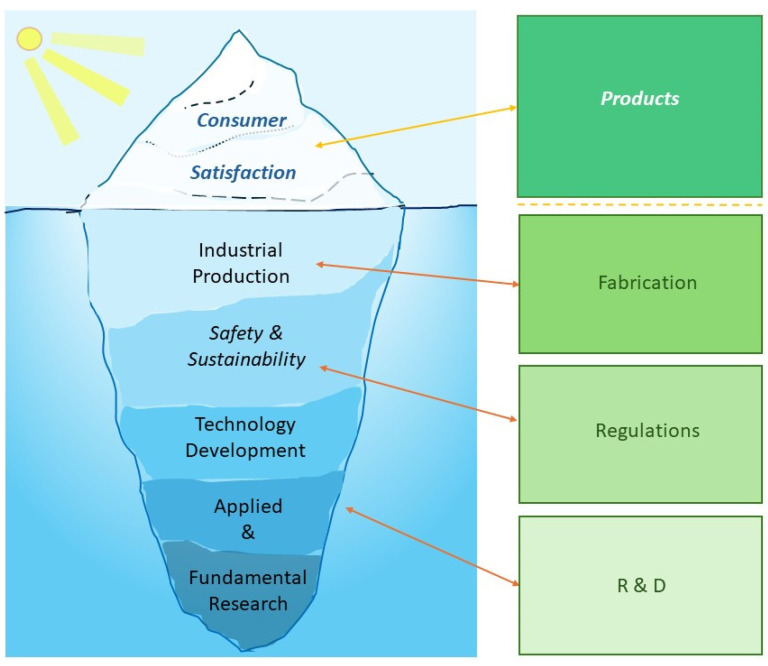
Overview of steps driving development in food packaging, shown as an iceberg (**left panel**), where consumer preferences need to be satisfied (transparently visible above the water level), while research, development, safety regulations, sustainability, and industry are shown below (often less visible/hidden under water level). Nanoarchitectonics is directly linked with the bottom blocks of the “iceberg”, but those blocks have direct relation with and influence its upper parts. Industrial production and governmental regulations accompanying development of products (**right panel**).

**Marketing**. As shown in Figure 6, consumers are the queens and kings, to whom the whole food chain is tailored to delivering good products for. But being at the tip of the iceberg, they often do not see the steps involved in the development of food packages. Food packaging represents a very important element; not only is it important to stores’ marketing, but it is also the last link in the food production chain linking food preparation with the consumers. Marketing is thus linked and associated with the packaging and can not only promote food for consumers [217] but also expose or bring adverse effects [218].

**Safety**. Safety of food is a multifold category. Food should be safe for consumers, and this is where food packaging takes the primary stand. The importance of this seemingly easy to handle aspect becomes apparent in waves; specifically, when a major outbreak of infection occurs, for example *E. coli* bacterial contamination, then this subject ultimately becomes the central topic of discussion among news outlets. With time and after reducing such contamination, food safety appears less regularly in the news and its subsistence is taken as an inherent norm. Sensors of food quality represent a very important evaluation tool, and together with the development of new materials, research and development of novel sensors represent growing areas.

In turn, food packaging should be safe for the environment, where attention falls on effects of materials used in food packaging on milieu. The presence of microplastic in the environment has been a known problem first for the environment and then ultimately for humans and animals. What is also quite peculiar is that the packaging industry constitutes the lowest product lifetime distribution, up to 3 years, among the eight most widely used industries including consumer and institutional products, textiles, electrical and electronic, transportation, industrial machinery, and building and construction.

Different international organizations [219], continents including Europe [220] and North America [221,222], and various countries [223,224,225,226] have carefully considered safety of food and food handling. Nanoscience and nanotechnology remain at the forefront of the next generation of food safety (https://www.efsa.europa.eu/en/topics/topic/nanotechnology, accessed on 23 February 2025), where nanoarchitectonics will play a very important role.

**Sustainability**. Pollution has been the central problem to overcome. And it is not only carbon dioxide and plastic pollution but also nitrogen pollution associated with the livestock industry that needs to be improved [227]. Various regulatory mechanisms are being developed, with the goal in mind being to construct sustainable and safe food production. Recent efforts in this area put focus on blue transformation initiatives aiming for sustainable, secure, and safe production in the coming years [228].

The development of new materials thus represents an essential component of developing sustainable packaging [56]. In this regard, new sustainability initiatives have been put in place in Strategic Framework 2022–2031 addressing the existing challenges [229]. Interaction with packaging materials [230] and the potential migration of chemicals into foods from packaging [231] and adhesives [232] represent essential health risks and challenges. Nanoarchitectonics has direct links with sustainable materials, since nanoscale properties determine all building blocks.

**Ethics and animal welfare**. Last but not least, in this section, we would like to discuss ethics which are applicable to animals with the aim of handling them with good practices. Assessing protocols for cattle [233] and working out a system for the evaluation of their stress [234,235] is important not only for the welfare of animals [236,237] but may also affect the quality of products [238]. Improving such practices can be achieved with good auditing [239].

Food packaging is an area closely linked with and driven by consumer–industry interaction, where the former eventually serves as the deciding figure in what will be researched and developed. The link between research, applied research, novel technological developments, industrial viability of their implementation, consumer satisfaction [240], and importantly, safety and sustainability, is seen in this area.

The outline of the different steps involved in producing packaging is shown in Figure 6. As can be seen from this figure, applied research and science represent a particularly important innovation axis, based on which the development of products follows. Largely driven by health and environmental regulations, protocols satisfying safety and sustainability regulations are directly coupled with this development, followed by industrial production. In Figure 6, all these steps are shown on the left-hand side, and they are shown as an underwater constituent of an iceberg that was shown to reflect the often-invisible components of lengthy and complicated processes of development. At the tip of the iceberg, consumers, the kings and queens, often do not see those underlying constituents of product development.

On the right-hand side of Figure 6, one can see the main building blocks depicted in green to indicate the development of sustainable products for consumers. The basic blocks of this part echo the underlying processes shown in the left panel.

## 8. Conclusions

It is almost impossible to bring food to the consumer without food packaging, which, although it increases the cost of products, is essential for protection. Here, we have provided the hierarchy of food packaging, distinguishing structural and functional properties, and emphasized the role of nanoarchitectonics in these areas.

Despite a variety of types of food (meat, fish, dairy, fruits, vegetables, gelled items, beverages, etc.) and different methods of their treatment, similarities exist between packaging methods regarding protection against bacteria, oxidation, light, temperature, and contamination whilst seeking methods that possess good mechanical properties for transportation, with an eye on preserving quality and extending shelf-life of foods for consumers. These similarities in protection by packaging exist and they can be linked through nanoarchitectonics, which determines the properties of materials and eventually packages at the nanoscale.

Furthermore, food packaging can adversely affect the environment because non-biodegradable microplastics were used in earlier packaging containers and boxes. That is why the development of new, green, and biodegradable materials is critical in this area. In the development of novel materials, originally used non-biodegradable fossil-based plastics (PE, PP, PET, PS, PVC, PA) are analyzed in contrast with novel fossil-based (PBS, PVA, PCL, PBAT) and bio-based biodegradable polymers, of which a large part represent polysaccharides. Edible biomaterials and hybrid and composite materials, as well as their assembly methods, are also discussed. Nanoarchitechnotics design and composition of these materials eventually determines the level of protection and sustainability of food products. It can be concluded that such advanced and sustainable materials and methods are necessary for food protection, with the aim of extending shelf life and providing safe, sustainable, recyclable, biodegradable, and economically effective and industrially viable solutions.

Finally, marketing, safety, sustainability, and ethics are also discussed here in the context of the overall supply chain, where the consumer and government regulations, on the one hand, and research and development driven by nanoarchitectonics on the other hand, will determine the food supply chain and shape the future of food packaging.

## Figures and Tables

**Table 1:** Common materials used for packaging of dairy products [58].

Dairy Product	Packaging Material
Liquid Milk	Polyethylene pouches (LDPE or LLDPE), paper board cartons (Tetra Pak or Tetra Brik), glass bottles, PET bottles.
Milk Powder	Flexible laminates (PET/BOPP/Al foil), tin container.
Ice Cream	Polypropylene containers, PET Laminates, paper board carton.
Butter	Parchment paper, wax-coated paper, cellophane, aluminum foil laminates, lacquered tin cans.
Cheese	Aluminum foil/paper laminates, cellophane/paper combinations, tinplate container.
Ghee	Polypropylene bottles, glass bottles, HDPE film pouches, tinplate container, polyethylene film pouches, LDPE/HDPE films.
Traditional Sweets	Aluminum foil, cellophane, HDPE films, LDPE films, PE laminate, tinplate container.

## Data Availability

No new data were created or analyzed in this study. Data sharing is not applicable to this article.
